# Prominent venous intravasation in hysterosalpingo-contrast sonography (HyCoSy): A case report

**DOI:** 10.1097/MD.0000000000047920

**Published:** 2026-03-06

**Authors:** Xia Tao, Xinyun Yang, Hongyun Zhang, Ping Li, Jiang Zhu

**Affiliations:** a Department of Ultrasound Medicine, Women’s Hospital School of Medicine Zhejiang University, Hangzhou, China; b Department of Reproductive Medicine, Women’s Hospital School of Medicine Zhejiang University, Hangzhou, China.

**Keywords:** case report, fallopian tube duplication malformation, hysterosalpingo-contrast sonography, venous intravasation

## Abstract

**Rationale:**

Hysterosalpingo-contrast sonography (HyCoSy) is a commonly used technique for assessing fallopian tube patency. A well-recognized complication of this procedure is venous intravasation (VI), which refers to the unintended entry of contrast medium into the uterine and parametrial venous systems. This highly variable complication often leads to misdiagnosis, which in turn compromises the subsequent management and treatment of affected patients.

**Patient concerns:**

A 32-year-old female who underwent a 4-dimensional HyCoSy for secondary infertility initially appeared to have a rare congenital defect, with images revealing three distinct tube-like structures resembling fallopian tubes, one on the right side of the uterus and the other two on the left side, which is suspicious for fallopian tube duplication malformation.

**Diagnoses:**

Real-time 2-dimensional imaging and dynamic cine-loop recordings revealed that the contrast agent originated from myometrial vessels rather than the tubal ostia. This confirmed a diagnosis of extensive VI mimicking fallopian tube duplication malformation, coupled with bilateral tubal obstruction.

**Interventions:**

The diagnosis was substantiated by the absence of peritoneal contrast spillage and the observation of delayed ovarian stromal enhancement, a sign of systemic recirculation. The findings were further verified via hysteroscopy and an intra-operative chromopertubation test. Following this, the patient was referred for in vitro fertilization-embryo transfer.

**Outcomes:**

After receiving appropriate fertility treatment based on the corrected diagnosis, the patient successfully achieved pregnancy via in vitro fertilization-embryo transfer.

**Lessons:**

Significant VI can manifest as organized “pseudo-tubular” structures that convincingly mimic congenital malformations. To ensure diagnostic precision, clinicians must track the anatomical origin of contrast egress and look for indirect signs of intravasation, such as delayed ovarian enhancement, to avoid unnecessary invasive procedures and guide correct clinical decision-making.

## 1. Introduction

Infertility is a significant global public health challenge, affects an estimated 186 million people of reproductive age worldwide.^[1]^ In the diagnostic workup of infertility, the assessment of tubal patency is a critical step, as tubal factors account for up to 40% of female infertility cases.^[2-4]^ Since Nannini et al first applied hysterosalpingo-contrast sonography (HyCoSy) to assess tubal patency in 1981, it has emerged as a first-line diagnostic tool for evaluating fallopian tube patency.^[5–7]^ Compared to traditional methods like hysterosalpingography and diagnostic laparoscopy, HyCoSy offers its advantages including the absence of ionizing radiation, minimal invasiveness, and cost-effectiveness, all while maintaining high diagnostic accuracy.^[8-11]^ Its utility is further enhanced by its compatibility with real-time imaging and the ability to store and retrospectively analyze dynamic data.^[12]^ The diagnostic reliability of HyCoSy is well-established in the literature. Multiple meta-analyses have demonstrated its high sensitivity and specificity, often exceeding 90%, particularly with the integration of 3/4-dimensional (3D/4D) ultrasound and the use of modern microbubble contrast agents.^[13-15]^ During HyCoSy, tubal patency is demonstrated by the unimpeded, non-tortuous flow of contrast medium through the fallopian tube. The definitive sign of patency is the subsequent spillage of contrast from the fimbria end, which typically manifests as periovarian fluid accumulation, often forming a characteristic “halo” around the ovary.^[10]^ This high level of accuracy has solidified HyCoSy’s role not only in infertility workups but also in assessing tubal status following recanalization procedures or in cases of suspected tubal pathology like salpingitis.^[16-18]^

Despite its diagnostic prowess, HyCoSy is not without limitations and potential pitfalls. A significant complication is venous intravasation (VI), defined as the unintended passage of contrast agent from the uterine cavity into the myometrial and pelvic venous systems, is a well-recognized complication and diagnostic pitfall of medical imaging.^[19,20]^ The classic sonographic appearance of VI is described as a diffuse, “reticular,” “lace-like,” “arborizing,” or “cloud-like” hyper echogenicity within the myometrium and para-uterine tissues, which often obscures the image and interferes with the assessment of tubal anatomy, potentially leading to diagnostic errors.^[19,21-23]^ Paradoxically, recent studies report a markedly higher incidence of VI in HyCoSy (13% to over 26%) compared to traditional hysterosalpingography (0.4% −7.2%).^[11,24,25]^ This phenomenon might reflect a unique interaction mechanism between microbubble contrast agents and the endometrial-myometrial junction (EMJ), a critical physiological barrier whose integrity can be compromised by conditions like adenomyosis or prior uterine instrumentation.^[16,24]^

Besides VI, other possible imaging dilemma such as false obstruction caused by fallopian tube spasm and poor visualization of the anatomical structures in the uterine cornual region would affect the accuracy of diagnosis as well.^[17,26]^ While VI is a recognized diagnostic challenge, its morphological presentations are typically diffuse and irregular. To our knowledge, the literature has few reports of VI forming such well-defined, large, and distinct “pseudo-tubular” structures that perfectly mimic a rare congenital anatomical malformation, fallopian tube duplication malformation (FTDM) on imaging. This atypical manifestation represents a novel and significant diagnostic pitfall with considerable clinical implications. Therefore, this report aims to delineate an unusual case where presented as such misleading pseudo-tubular structures. By analyzing the key sonographic features and dynamic processes that guided the correct diagnosis, we seek to equip clinicians with the necessary knowledge to recognize the pitfall, thereby preventing misdiagnosis and subsequent unnecessary invasive interventions.

## 2. Case presentation

This case report was prepared in accordance with the CARE guidelines.^[27]^ Written informed consent was obtained from the patient.

### 2.1. Patient information

The patient was a 32-year-old woman presenting with secondary infertility. She had a history of one full-term spontaneous vagina delivery 5 years ago. Her notable past medical history included polycystic ovary syndrome, but follicular monitoring had confirmed regular ovulation. There was no history of uterine surgery, pelvic inflammatory disease, or endometriosis, and her husband’s semen analysis was also normal.

### 2.2. Clinical findings

**Figure 1.**
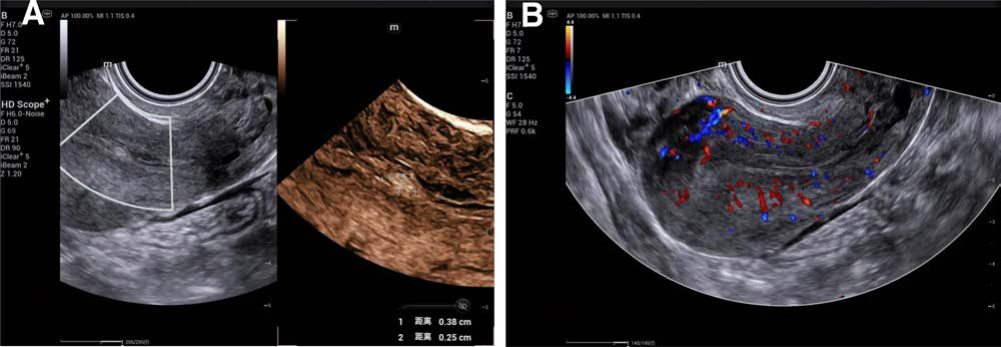
Pre-procedural transvaginal ultrasound. (A) A 4 mm strongly echogenic polypoid structure is detected in the lower segment of the uterus. (B) No other abnormalities were detected in the uterus.

A routinely pre-procedural transvaginal ultrasound identified a polypoid echogenic focus measuring approximately 4 mm in the lower uterine segment (Fig. 1). No remarkable area was spotted at myometrium, ovaries, and adnexal regions. Routine tests for leukorrhea and urine human chorionic gonadotropin were negative.

### 2.3. Timeline

A timeline of the patient’s diagnosis and treatment included in this report are summarized in Table 1 below.

**Table 1:** Timeline of the patient’s diagnosis and treatment.

Time	Event
Initial visit	Patient presents with secondary infertility
Pre-procedural assessment	A routine transvaginal ultrasound is conducted, which identifies a 4 mm polypoid echogenic focus in the lower uterine segment
Day 4 post-menstruation	The patient undergoes a 4D hysterosalpingo-contrast sonography examination - Initial imaging raises suspicion of fallopian tube duplication malformation. - A detailed review of dynamic imaging corrects the diagnosis to bilateral tubal obstruction with prominent venous intravasation
Subsequent to HyCoSy	The patient undergoes a hysteroscopic polypectomy - A methylene blue hydrotubation is performed - Confirms the diagnosis of bilateral tubal obstruction
Follow-up	The patient is referred to a reproductive medicine center for assisted reproductive technology and successfully conceives

### 2.4. Diagnostic assessment

#### 2.4.1. *Examination* method

Equipment and parameters: The examination was performed using Nuewa A20 ultrasound system (Mindray, Shenzhen, China) equipped with an SDE10-2WU endocavity volume probe, utilizing the system’s 3D/4D HyCoSy and contrast-enhanced harmonic imaging modes. Before the contrast examination, a routine 2-dimensional ultrasound scan (AP = 100%, MI = 1.1, TIS = 0.4) was performed to assess the pelvic condition and the positional relationship between the bilateral ovaries and the uterus. Subsequently, a 3D (AP = 100%, MI = 1.4, TIS = 0.117) prescan is performed, which should include the uterus and bilateral ovaries, to determine the scanning depth of the images. The Contrast 4D mode (AP = 8.32%, MI = 0.308, TIS = 0.041) was used for full-process video recording during the injection of the contrast medium. Finally, the Contrast dual real-time mode (AP = 3.16%, MI = 0.137, TIS = 0.006) was applied to conduct a comprehensive examination of reevaluate the distribution of the contrast agent within the pelvic cavity.

Contrast agent: The procedure used Lidaxin™, a second-generation ultrasound contrast agent composed of perfluoropropane gas microspheres encapsulated in human albumin.^[28]^ Following the manufacturer’s protocol, the agent was prepared by reconstituting 0.5 g of lyophilized powder in 3 mL of normal saline. This stock solution was then drawn into a 20-mL syringe and further diluted with normal saline to a final volume of 20 mL for administration.

Technical operation: The procedure was performed day 4 post-menstruation. After the patient emptied her bladder and, a 12-French double-lumen catheter was gently inserted into the uterine cavity after the patient was placed in the lithotomy position, and its balloon was inflated with 1 mL of saline solution to secure its position. The diluted contrast suspension was then slowly and steadily injected under manual control, with careful attention to avoiding excessive injection pressure to minimize any sharp increase in intrauterine pressure and potential damage to the endometrium.

#### 2.4.2. Imaging findings and diagnostic reasoning

**Figure 2.**
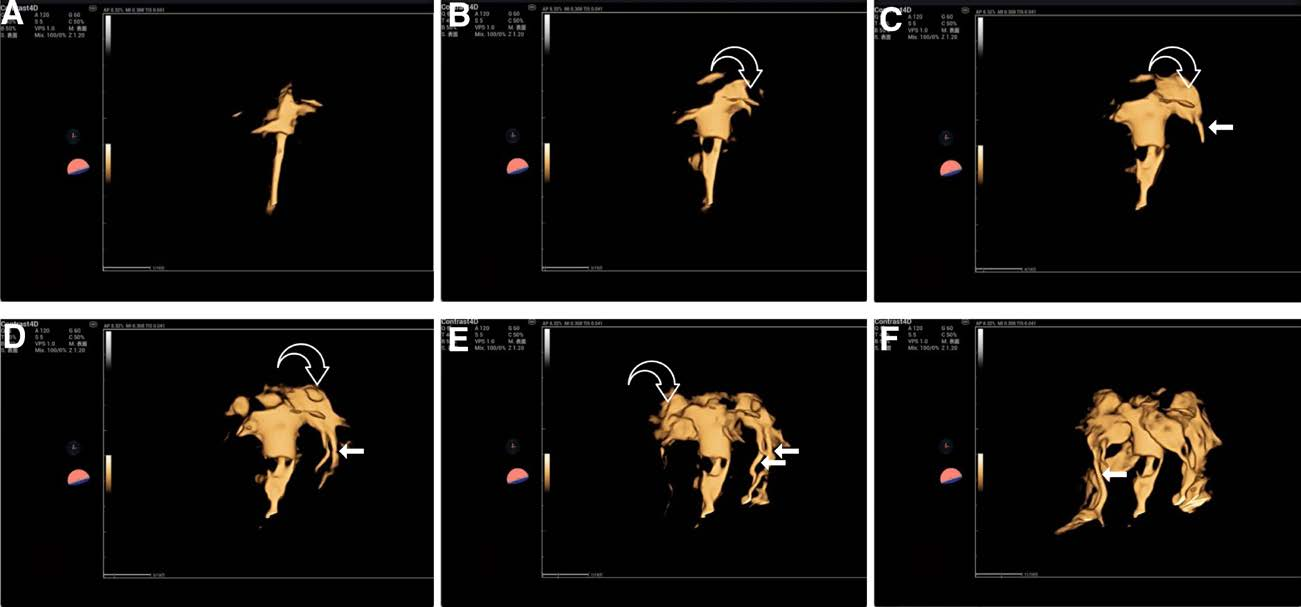
4D HyCoSy mode: (A) the contrast agent enters the uterine cavity along the contrast catheter. (B) The left uterine horn begins to visualize, but its morphology is abnormal (the white hollow arrows). (C) The contrast agent further fills and distends the left uterine horn (the white hollow arrows), with a tubular structure visible at its distal end (the white solid arrows). (D) Two tubular structures are visible at the margin of the distended left uterine horn (the white solid arrows). (E) The left tubular structure continues to extend, showing an appearance highly like that of a fallopian tube-like structure (the white solid arrows); meanwhile, the myometrium of the right uterine horn exhibits abnormal visualization and distension (the white hollow arrows). (F) A fallopian tube-like structure is visible at the margin of the distended right uterine horn, which is like the left tubular structure (the white solid arrows). 4D = 4-dimensional, HyCoSy = hysterosalpingo-contrast sonography.

**Figure 3.**
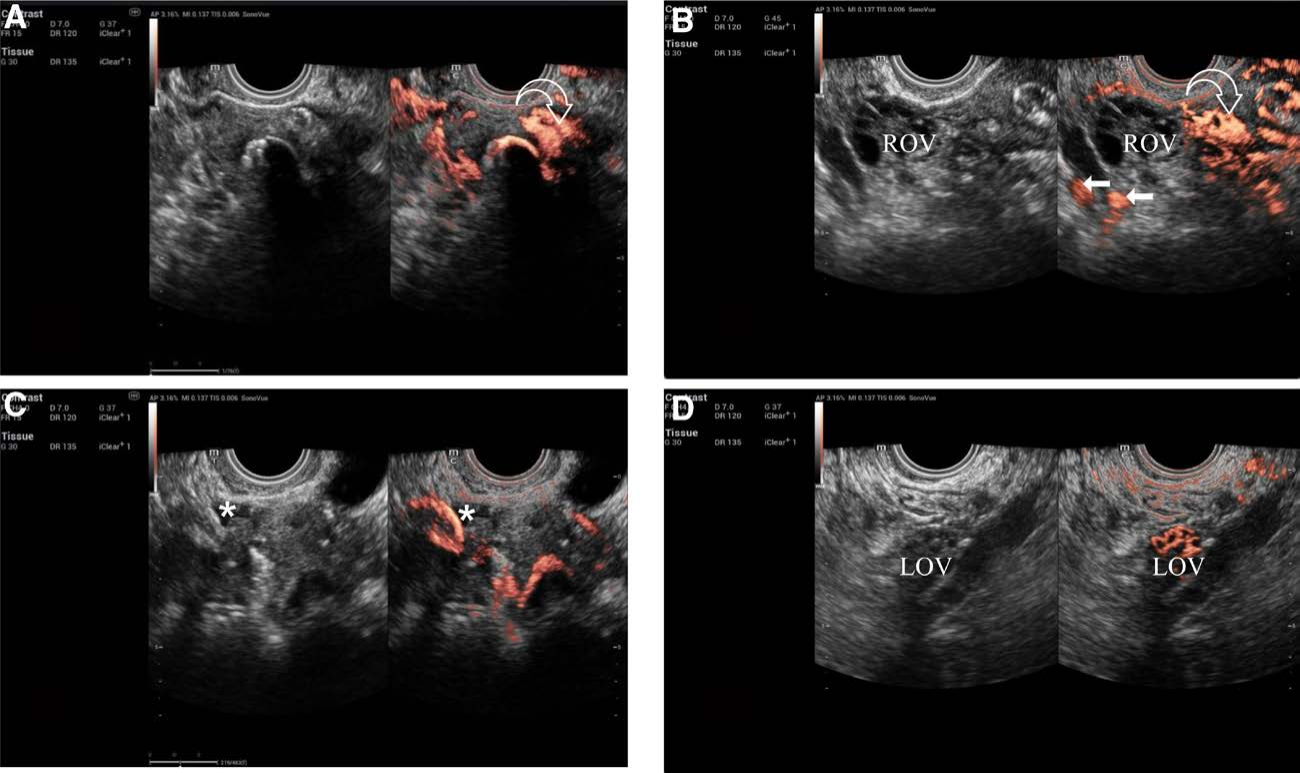
2D contrast mode: (A) the contrast agent is visualized in the myometrium (the white hollow arrows). (B) No contrast agent was observed around the right ovary (labeled as “ROV”), the contrast agent is visualized in the right iliac vein (the white solid arrows) and myometrium (the white hollow arrows). (C) No contrast agent is visualized in the pelvic spaces (the white asterisk). (D) Delayed imaging showed contrast agent perfusion in the parenchyma of the left ovary (labeled as “LOV”). 2D = 2-dimensional, LOV = left ovary, ROV = right ovary.

Initial imaging presented a significant diagnostic dilemma. As shown in Figure 2, upon injection of the contrast agent, 2 well-defined, regular, tubular hyperechoic region was observed in the left para-uterine region, with a similar pattern presenting subsequently at the right uterine cornu, forming a tri-tubular structure, resembling fallopian tubes on ultrasound imaging, prompting an initial differential diagnosis of FTDM. While 3 well-defined, tubular structures resembling FTDM were observed, the concurrent absence of peritoneal spillage suggested bilateral tubal obstruction (Fig. 3). A systematic, multiplanar, frame-by-frame review of the 4D dynamic cine-loop was critical in resolving this contradiction. This detailed analysis revealed the definitive finding: the contrast agent originated not from the tubal ostia, but as diffuse extravasation directly from the myometrium. This confirmed the structures were dilated parametrial venous plexuses resulting from extensive VI. This diagnosis was further substantiated by the complete lack of a periovarian halo or distal spillage into the pelvic cavity. Conclusive physiological proof of VI was obtained minutes later, when a diffuse, uniform signal enhancement appeared within the ovarian parenchyma. This delayed ovarian enhancement is a specific sign of systemic recirculation, indicating the contrast agent had entered the venous system, passed through the cardiopulmonary circulation, and returned to the ovaries via arterial flow. This evidence definitively confirmed VI and eliminated any possibility of tubal patency, thus resolving the initial ambiguous findings.

#### 2.4.3. Therapeutic intervention and follow-up

Based on the definitive HyCoSy diagnosis of bilateral tubal obstruction and extensive VI, alongside a pre-procedurally identified endometrial polyp, the patient was advised to undergo a hysteroscopic polypectomy. During this procedure, an intra-operative chromopertubation test was performed by injecting diluted methylene blue through the operating channel. This test confirmed the initial diagnosis, as no dye effluent was observed from either tubal ostium; instead, significant resistance and reflux from the uterine cavity were noted. Consequently, the patient was referred for fertility treatment and subsequently successfully conceived following in vitro fertilization-embryo transfer.

## 3. Discussion

To our knowledge, this case report is the first to document a highly atypical presentation of VI during HyCoSy, where organized, linear “pseudo-tubular” structures convincingly mimicked the sonographic appearance of a rare congenital anomaly, FTDM. The definitive diagnosis of bilateral tubal obstruction with extensive VI was established through a systemic sonographic analysis and later confirmed by hysteroscopy. This finding not only highlights a novel and clinically significant diagnostic pitfall but also underscores the need for deeper investigation into the pathophysiology, varied presentations, and differential diagnostic strategies for VI to prevent misdiagnosis.

### 3.1. The diagnostic pitfall: differentiating VI from FTDM

FTDM is an extremely rare Müllerian duct anomaly with such a low incidence that it is not included in major classification systems like the American Society for Reproductive Medicine 2021 guidelines.^[29]^ Based on limited case reports, the imaging hallmark of FTDM should be 2 distinct, smooth-walled tubular structures originating from the uterine cornu, following the expected anatomical course of a fallopian tube, and demonstrating fimbrial structures with peritoneal spill if patent.^[30,31]^ Its embryological origin is thought to involve the splitting of the cranial end of the Müllerian duct or abnormal development of the paramesonephric ducts.^[32]^ 3D ultrasound and MRI are considered valuable for diagnosis due to their ability to clearly delineate the external uterine contour and the origin of the tubes.^[33,34]^ Despite a highly deceptive initial presentation on imaging, an accurate diagnosis was achieved through a systematic workflow. To provide a practical clinical tool, the key differentiator between VI and genuine tubal anomalies (e.g., FTDM) are summarized below (Table 2).

**Table 2:** Summary of key differences between VI and genuine tubal anomalies (e.g., FTDM).

Feature	Prominent venous intravasation (VI)	True tubal anomaly (e.g., FTDM)
Origin of flow	Emerges directly from the myometrium; “source-tracking” shows no connection to the tubal ostia	Originates from the uterine cornua, manifesting as a direct, linear continuation extending from the endometrial cavity
Morphology	Often tortuous with irregular caliber; follows vascular anatomy, not necessarily coursing toward the ovary, lacks a distinct structural wall	Smooth-walled with a relatively uniform caliber; follows the normal anatomical course of a fallopian tube
Endpoint	No peritoneal spill. Contrast disappears after draining into larger pelvic veins (washout)	If patent, shows fimbrial spill, a periovarian halo, and free fluid in the pelvis
Systemic signs	Specific sign: Delayed enhancement (2–3 min) of ovarian parenchyma due to systemic recirculation	None: No systemic recirculation of contrast agent occurs

VI = venous intravasation, FTDM = fallopian tube duplication malformation.

### 3.2. Pathophysiology of prominent venous intravasation: a proposed mechanism

EMJ, also referred to in imaging literature as the junctional zone, is the critically important and histologically distinct interface between the inner mucosal layer of the uterus (endometrium) and the outer smooth muscle layer (myometrium).^[35,36]^ Therefore, it remains the core of this barrier, and its integrity is paramount. Literature suggests that the EMJ is a functionally distinct zone whose integrity can be compromised by various factors, such as adenomyosis, prior uterine instrumentation, or childbirth trauma, creating potential pathways for fluid or contrast to permeate from the cavity into the myometrium.^[35-39]^ Here, we propose a “two-hit” pathophysiological model-a preexisting compromised EMJ combined with high intrauterine pressure from tubal obstruction-to explain this unusual manifestation.

The first hit was the compromised integrity of the EMJ barrier. The occurrence of VI is fundamentally a breakdown of the physiological barrier between the uterine cavity and the myometrial vascular system. The EMJ is the core of this barrier, and its integrity remains crucial.^[35,38]^ Previous research indicates that the EMJ is a functionally unique zone whose structural integrity can be compromised by various factors, thereby creating a potential pathway for fluid or contrast medium to intravasate from the uterine cavity into the myometrium.^[37,38]^ This particular patient presented with several risk factors known to weaken the EMJ, including potential micro-trauma from a previous delivery, local endometrial disruption from a polyp,^[11,21]^ and a thin endometrium (5 mm) due to the early timing of the procedure – a confirmed independent risk factor for VI.^[23,40]^

The second hit was an obstruction-induced, pressure-driven shunting of the contrast agent, acting upon the already compromised EMJ barrier.^[41]^ Tubal obstruction effectively creates a closed hydraulic system. As the contrast is injected, it cannot exit through the fallopian tubes, causing a rapid and significant rise in intra-uterine pressure.^[42]^ When this pressure exceeds the resistance of the endometrial-myometrial interface and the intravascular pressure of the myometrial veins, the contrast agent is forced to seek an alternative path of least resistance: shunt through the impaired EMJ into the low-pressure myometrial venous plexus.^[43]^ The resulting high-volume, high-velocity influx, classified as a moderate-to-severe event, opacified the larger-caliber parametrial veins.^[24]^ This created the well-defined, pseudo-tubular structures that ultimately mimicked the appearance of fallopian tubes.^[44]^

### 3.3. Interpreting “Painless” VI

Notably, while VI is often linked to pain from elevated intrauterine pressure secondary to tubal obstruction, this case demonstrates that extensive VI can occur^[42]^ We speculate that the underlying bilateral tubal obstruction may paradoxically prevent. Discomfort by shunting pressure into the low-resistance venous system, thereby avoiding significant intrauterine pressure accumulation. Consequently, the absence of pain is an unreliable indicator for excluding significant VI. Furthermore, we propose that significant, asymptomatic VI is not merely an iatrogenic event or a consequence of obstruction, but an independent pathophysiological marker. It may indicate a compromised EMJ barrier, a defect associated with other infertility factors like adenomyosis and implantation failure.^[35,36,45]^ Therefore, observing this phenomenon offers diagnostic insights that extend beyond the primary assessment of tubal patency.

### 3.4. Clinical implications of recommendations

This case provides several actionable recommendations for clinicians. For sonographers and gynecologists performing HyCoSy, VI must be a primary differential diagnosis when multiple “tube-like” structures are spotted. When anomalous tubular structures are visualized during HyCoSy, a systematic evaluation is critical to differentiate them from true fallopian tubes. Operators must first identify the initial egress point of the contrast agent, determining if it originates from the anatomically correct tubal interstitium or as diffuse extravasation from the myometrium. Furthermore, the morphology of these pseudo-tubular structures should be assessed, as venous plexuses resulting from VI are typically more irregular, follow a tortuous course, and may not be oriented toward the ovary. In contrast, the definitive evidence of true tubal patency is the observation of fimbria spillage or a periovarian halo in the pelvic cavity, a sign that is conclusively absent in VI.^[10]^ Lastly, examining for other specific signs of intravasation is encouraged to confirm the identity of the enhanced signals.

### 3.5. Analysis of misinterpretation risks

Misinterpreting this imaging artifact poses 2 significant clinical risks. The more critical error is a false-negative diagnosis, where VI is mistaken for tubal patency in a patient with bilateral obstruction, which can lead to fruitless therapies and critically delay access to Assisted Reproductive Technology. Conversely, a false-positive diagnosis, where VI is misidentified as an anatomical anomaly, can provoke patient anxiety and prompt unnecessary invasive investigations. Therefore, to mitigate these risks, minimizing VI incidence requires adherence to best practices, such as scheduling the procedure during the mid-follicular phase (Days 7–11) and utilizing a slow, low-pressure injection technique.^[23]^

### 3.6. Strengths and limitations

To our knowledge, this is the first report to detail a case where the sonographic appearance of VI directly mimicked FTDM, thereby highlighting a novel and clinically significant diagnostic pitfall. Through an in-depth analysis of the pathophysiology and imaging features, it provides valuable differential diagnostic insights for clinical practice. However, as a single case report, the findings cannot be generalized. The strength of this study lies in its high educational value, as it highlights a rare but clinically significant diagnostic error with major implications for patient management. However, as a single case report, its findings cannot be generalized. The academic value of this study lies in its educational significance: it not only clarifies the key diagnostic points in HyCoSy but also exerts a profound impact on patient management – misdiagnosis could lead to patients receiving inappropriate treatment.

## 4. Conclusion

This case underscores that prominent VI, a common but highly variable complication of HyCoSy, can create significant diagnostic traps by atypically manifesting as organized “pseudo-tubal” structures. Therefore, clinicians must maintain a high index of suspicion regarding the diverse sonographic presentations and pathophysiology of VI. Moreover, the value of indirect evidence should be emphasized, as signs like the absence of peritoneal spillage and delayed ovarian stromal enhancement are powerful indicators that can confirm VI while excluding tubal patency and must be actively sought during the examination. In cases of ambiguous imaging, a rigorous application of advanced techniques, particularly 4D dynamic imaging to track the source of contrast outflow, is critical for an accurate differential diagnosis, which in turn prevents unnecessary interventions and guides correct clinical decision-making.

## Author contributions

**Data curation:** Xinyun Yang.

**Investigation:** Hongyun Zhang.

**Methodology:** Ping Li.

**Writing – original draft:** Xia Tao.

**Writing – review & editing:** Jiang Zhu.
